# Moving toward rice self-sufficiency in sub-Saharan Africa by 2030: Lessons learned from 10 years of the Coalition for African Rice Development

**DOI:** 10.1016/j.wdp.2021.100291

**Published:** 2021-03

**Authors:** Aminou Arouna, Irene Akoko Fatognon, Kazuki Saito, Koichi Futakuchi

**Affiliations:** Africa Rice Center (AfricaRice), 01 BP 2551 Bouake 01, Bouake, Cote d’Ivoire

**Keywords:** Rice, Demand-pull factors, Supply-push factors, Sustainable investments, Self-sufficiency, Sub-Saharan Africa

## Abstract

The Coalition for African Rice Development (CARD) was initiated as a policy framework with the aim of doubling rice production in sub-Saharan Africa (SSA) over the period from 2008 to 2018. This paper assesses the contribution of the CARD policy to rice production and forecasts the local rice supply and demand to provide a better understanding of the policies needed to attain rice self-sufficiency by 2030. A combination of the autoregressive integrated moving average method and counterfactual approach was adopted using rice statistical data from 23 countries in SSA. The results showed that the contribution of CARD to paddy rice production in 2018 was 10.2 million tons, equivalent to 74% of the target. This contribution resulted from increases in area and yield of 23% and 19%, respectively. However, the yield growth rate was not sustainable in almost two-thirds of countries. Investments in supply-push factors such as fertilizer and irrigation development, which were the focus in the past, have limited effects on rice production. We conclude that sustainable investments in demand-pull factors such as the private-led modern milling sector and contract farming development should be prioritized to achieve rice self-sufficiency in SSA.

## Highlights

• Contribution of the CARD policy to rice production in sub-Saharan Africa.
• Forecasts local rice supply and demand by 2030.
• Significant impact of the CARD policy was found on local production.
• Yield growth rate was not sustainable in most countries throughout the CARD period.
• Demand-pull factors development should be prioritized over the supply-shift actions.

## 1 Introduction

Rice is an important staple crop that plays an important economic role and feeds approximately half the world’s population (Fahad et al., 2019). Global rice consumption in 2018 was estimated at more than 488 million tons (MT) (USDA, 2019), with Asia accounting for 90% of the production and consumption. However, rice consumption is increasing rapidly in sub-Saharan Africa (SSA).

In SSA, rice consumption exceeds production. In 2018, rice consumption in SSA was estimated to be approximately 33.2 MT of milled rice, which was partially fulfilled by the importation of approximately 15.5 MT, an amount equivalent to 33% of that traded in the world market (USDA, 2019). The estimated import bill of the rice was US$ 6.4 billion in 2018. In SSA, rice is fundamental for food security and social stability (Arouna et al., 2017, Seck et al., 2010). Its consumption is increasing more rapidly than any other commodity and is driven by the triple effect of population growth, urbanization and changing of consumer behavior in the region. Demand for rice has increased at a rate of 6% per annum over the last ten years (USDA, 2019), giving it the fastest growth rate in the world. In addition, its contribution to food energy is increasing, while this contribution is decreasing for certain cereals, such as millet and sorghum.

The increasing role of rice in the food basket of consumers has made it a political crop in the sense that its price and accessibility influence social stability (Seck et al., 2010). With the 2007–2008 food crisis, there was a threefold increase in the world price of rice within a few weeks, and the average global rice price has not returned to its pre-2007 level (Soullier et al., 2020). According to the agricultural outlook for the 2019–2028 period, rice imports are expected to be high in SSA (OECD/FAO, 2019), and world milled rice prices are expected to increase to US$ 470 per ton by 2028 compared to US$ 447 in 2018. The increase in rice prices coupled with the increase in imports will result in higher import bills in the region. However, high potential exists in Africa to close the gap between demand and supply through increases in domestic rice production (van Oort et al., 2015) and, thus, to achieve the objective of rice self-sufficiency promoted by different policy makers (Clapp, 2017).

As part of policy makers’ and international communities’ efforts to strengthen the rice sector to promote self-sufficiency in SSA, a policy framework known as the “Coalition for African Rice Development” (CARD) was launched during the Fourth Tokyo International Conference on African Development. The CARD policy framework aimed to double rice production in SSA countries between 2008 and 2018. The CARD policy framework was implemented in links to existing programs such as the Comprehensive Africa Agriculture Development Program (CAADP), the New Partnership for Africa’s Development (NEPAD) and the Alliance for a Green Revolution in Africa (AGRA) (Golay, 2010).

This paper aims to assess the contribution of the CARD policy framework to rice production over the period 2008 to 2018 of implementation and to forecast local rice supply and demand to provide a better understanding of the policy measures necessary to achieve rice self-sufficiency in the region by 2030. This study therefore seeks to answer the following research questions: (i) What is the contribution of CARD policy to rice production in SSA? (ii) What are the determinants of CARD's impact on rice production? What are the yield and area growth rates required to achieve rice self-sufficiency in SSA by the target date of the Sustainable Development Goals (SDGs) in 2030? Although the impact of technologies is frequently discussed in the literature (Arouna et al., 2017, Jiao and Schneeberger, 2017, Kassie et al., 2011, Saito et al., 2019), the impact assessment of policy measures is limited, and to the best of our knowledge, this is the first attempt to estimate the impact of the CARD framework. In addition, while extensive literature exists on the separate application of the autoregressive integrated moving average (ARIMA) model (Ruby-Figueroa et al., 2017, Sen et al., 2016) and counterfactual approach (Arouna et al., 2017, Manda et al., 2019), the combination of the two methods to assess the impact of policy is uncommon. Indeed, the ARIMA model can help generate the counterfactual situation, which is essential in the counterfactual approach for impact assessment. The contributions of the paper to the literature are therefore threefold. First, we attempt to estimate the real contribution of policy measures to rice production. We also analyze the determinants of the impact of CARD by considering both demand-pull and supply-push factors. In fact, the National Rice Development Strategies (NRDS) plan considered both demand-pull and supply-push investments. However, the focus was mainly on supply-push investments (Demont, 2013). Therefore, it is important to analyze the contribution of this policy orientation. Second, this paper contributes to filling a research gap by using a combination of the ARIMA model and counterfactual approach to assess the impact of the CARD framework. Third, to determine the effort needed to achieve self-sufficiency in SSA, we forecasted rice production and consumption by 2030. Findings from this study will inform recommendations for policy makers and future research aimed at achieving rice self-sufficiency in SSA.

## 2 Overview of the CARD

**Fig. 1:**
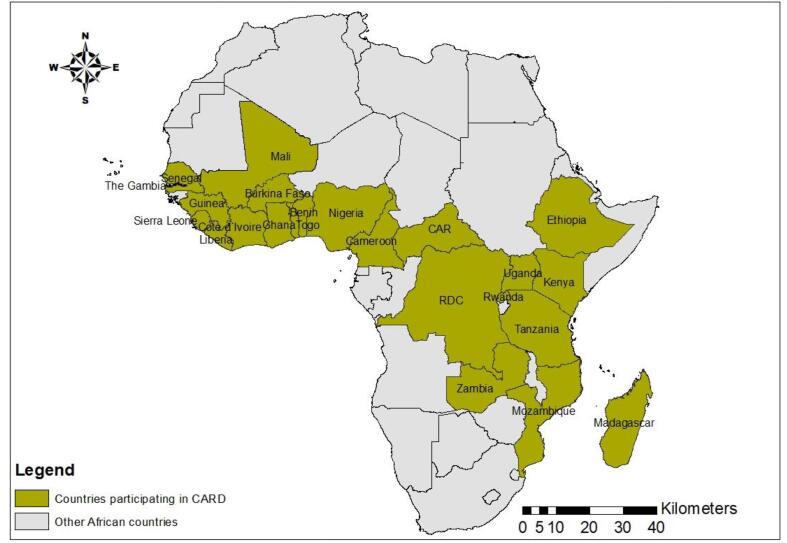
Africa map with CARD Phase 1 participating countries.

In relation to the 2007–2008 food crisis, when the export price of rice exceeded US$ 1000 per ton (Pandey et al., 2010), rice sector development in SSA became crucial for food security. To close the local rice supply and demand gap, the CARD policy framework was initiated in 2008 to promote rice sector development in 23 countries in SSA (Fig. 1). The main goal of the CARD policy framework was to double rice production, from 14 MT of paddy rice to 28 MT between 2008 and 2018 in SSA. To boost rice production, participating countries developed the first generation of NRDSs (Demont, 2013) as policy documents for rice development in each country. An NRDS is a comprehensive strategy for achieving the rice development goal in a country. The formulation of the NRDSs was led by national institutions and subjected to a broad policy-based dialog and consultation with the active participation of relevant stakeholders in the rice value chain. Each NRDS analyzed the entire rice sector in the country, and the focus was on short-, medium- and long-term actions. As a result, 23 African countries have subsequently developed NRDS documents that are available on the CARD web portal^1^. The NRDSs were designed to respond to different challenges in the rice value-chain development, such as the lack of appropriate policies, weaknesses in policy research and planning for increased rice production, the lack of availability of and access to quality seeds and production inputs (fertilizers, herbicides, etc.), the lack of irrigation schemes, difficulties in water management and weakness in agricultural extension systems. Although the major focus of the NRDSs was on investments for increased production, some investments on value-adding technologies, infrastructure and value-chain upgrading were noted (Demont, 2013). Through the implementation of the NRDSs, the main actions taken to achieve the CARD goal were (1) yield-enhancing technical package distribution, (2) on-farm demonstration plots, (3) the introduction and distribution of small- and medium-scale rice processing equipment, (4) high-level advocacy measures, and (5) partnerships between stakeholders (JICA, 2018). Many projects were implemented during the decade (2008–2018) in relation to the NRDSs. These projects were generally part of the countries’ programs on food security, such as Benin’s Strategic Plan for the Revival of the Agricultural Sector, Côte d’Ivoire’s recovery plans for rice and other food crops, Mali’s Rice Initiative, Nigeria’s Agricultural Transformation Agenda and Senegal’s Great Agricultural Offensive for Food and Abundance. Between 2008 and 2018, 218 projects across the 23 countries were supported by various partners, including the United Nations Food and Agriculture Organization (FAO), the World Bank, the African Development Bank (AfDB), the United States Agency for International Development (USAID), the Africa Rice Center (AfricaRice) and the Japan International Cooperation Agency (JICA). The total investments in the projects are estimated to be approximately US$ 9 billion (CARD, 2019)^2^. With achievement of the set target, CARD entered its second phase in 2019, with a new target of further doubling annual rice production in SSA, from 28 MT to 56 MT, by 2030. Nine countries, two development partners and five regional economic communities newly joined the initiative.

## 3 Methodology

### 3.1 Impact assessment approach

The CARD aimed to increase rice production from 14 MT in 2008 to 28 MT in 2018. To assess the level of achievement of this objective, the trends in rice yield, area and production from 2008 to 2018 were calculated. The growth rates of the decades before and after the 2007–2008 food crisis (1996–2007 and 2008–2018) were also computed and analyzed.

Although the trend analysis allowed us to assess the achievements of the CARD policy objective, it did not allow us to quantify the impact or net effect of the CARD on rice production statistics (production, yield and area) and self-sufficiency. To estimate the impact of the CARD, we used the counterfactual framework. The true impact of the CARD is the difference between the observed situation (situation with CARD) and the situation that would have existed if the CARD policy framework had not acted (counterfactual situation). The counterfactual situation represents the status of a country had it not participated in the CARD. However, the availability of counterfactual data to estimate the net effect of an intervention is often the most challenging part of impact analysis. One cannot observe the counterfactual situation of the CARD countries because all countries participated through differing actions. To address this known missing component of the data in the counterfactual framework (Anderson et al., 2016), we simulated the counterfactual situation.

We employed the ARIMA model, which is a time series model largely used in the literature to forecast the future or to predict missing values (Hassani et al., 2009, Wang et al., 2018). Similar estimation of counterfactual values was used by Wilson and Ren (2007, p. 1659) to estimate the simulated exchange rate regimes and compare them with actual regimes in Asian countries. The ARIMA model performed similarly in forecasting as other models, such as the singular spectrum analysis technique and the Holt–Winters model (Hassani et al., 2009). Using the ARIMA model, we generated the counterfactual situation by forecasting the values of the yield and area from 2008 to 2018 based on the historical trends from 1960 to 2007^3^.

#### 3.1.1 Yield and area impact estimation model

The impacts of the CARD on area and yield in yeart were expressed as follows:

(1) $$\nabla A_{\mathrm{It}}=A_{\mathrm{ob},t}-A_{\mathrm{cf},t}$$

(2) $$\nabla Y_{\mathrm{It}}=Y_{\mathrm{ob},t}-Y_{\mathrm{cf},t}$$

where $\nabla A_{\mathrm{It}}$ and $\nabla Y_{\mathrm{It}}$ denote the impact of the CARD policy on area and yield in yeart, respectively. $A_{\mathrm{ob},t}$ and $Y_{\mathrm{ob},t}$ represent the observed value of the area and yield in year t, respectively, and $A_{\mathrm{cf},t}$ and $Y_{\mathrm{cf},t}$ are the counterfactual situation of the area and yield in yeart in the absence of implementation of the CARD.

#### 3.1.2 Production impact estimation model

The change in rice production can occur through the increase of the rice productivity per unit of land and through the expansion of the rice harvested area (You et al., 2011). The impact of the CARD on production could be related to (i) a change in yield only, (ii) a change in area only, or (iii) a contribution to changes in yield and area. Therefore, the impact on production was derived as follows (for simplicity, the index *t* is not spelled out):

(3) $$\nabla P=\left(A_{\mathrm{cf}}*\nabla Y\right)+\left(\nabla A*\nabla Y\right)+\left(Y_{\mathrm{cf}}*\nabla A\right)$$

Using $A_{\mathrm{ob}}=A_{\mathrm{cf}}+\nabla A$ from Eq. (1), Eq. (3) is equivalent to

(4) $$\nabla P=A_{\mathrm{ob}}*\nabla Y+\left(Y_{\mathrm{cf}}*\nabla A\right)$$

where ∇P is the impact on rice production and the other parameters are as defined in Eqs. (1), (2).

The determinants of the impact of CARD on paddy production were modeled using simple ordinary least squared regressions. Two categories of variables were considered: supply-push factors and demand-pull factors. Four variables were considered supply-push factors in the final model: number of varieties released or adopted, fertilizer used per hectare, number of extension agents and share of irrigated area. Importance of investments on value-chain upgrading and dominance of preference for local rice were considered demand-pull factors. For the variable “importance of investments on value-chain upgrading”, we used the classification of Soullier et al. (2020), who defined three groups of countries based on the importance of investments for value-chain upgrading: a group with high or dynamic value-chain upgrading investment, a group with moderate value-chain upgrading investment and a group with no evidence of value-chain upgrading investment. West African countries were classified as in Soullier et al. (2020) while other countries were classified based on expert opinion (see Table A1 in Appendix). For the variable “dominance of preference for local rice”, we used the classification of Demon (2013), who defined three groups of countries: coastal countries with dominant preference for local rice, coastal countries with dominant preference for imported rice and landlocked countries (Table A1 in Appendix). Other variables, including number of projects focusing on either seed and paddy production or postharvest, were considered, but they did not improve the quality of the model. Similarly the dummy variables were removed because of high correlation with the variables “coastal countries with dominant preference for local rice” and “coastal countries with dominant preference for imported rice”. The dependent variable is the cumulative impact over the CARD period 2008–2018.

#### 3.1.3 Self-sufficiency impact estimation approach

The production to consumption ratio was used as an indicator of self-sufficiency (van Oort et al., 2015). We calculated the impact on self-sufficiency in terms of the annual contribution of the CARD to self-sufficiency. The annual contribution of the CARD to self-sufficiency is given by the following equation:

(5) $$\nabla SF=\left(\frac{\nabla P}{C_{\mathrm{ob}}}*100\right)$$

where ∇SF is the impact of the CARD on rice self-sufficiency, $C_{\mathrm{ob}}$ is the observed consumption, and ∇P is the impact on rice production.

### 3.2 ARIMA model estimation procedure

In forecasting, the ARIMA model is a commonly used approach (Wang et al., 2018). The ARIMA model is a time series model that forecasts variables. The model uses information from the variable itself to forecast its trend. Each variable in the series is forecasted by using its historical values. To fit a time series ARIMA model, stationarity is a necessary condition. The stationarity of the time series implies that the mean and variance of the series are constant. When the time series is nonstationary, it is differenced to make it stationary. After the stationarity of the series is verified, the ARIMA fitting model is run to identify the stochastic process of the time series and to forecast the future values accurately. The process is referred to as ARIMA (p, d, q), where p and q are the order of the autoregressive (AR) and moving-average (MA) models, respectively, and d refers to the order of differencing required to make the series stationary. The ARIMA model follows four steps: identification, estimation of parameters, diagnostic checking and forecasting (Hassani et al., 2009, Wang et al., 2018).

### 3.3 Forecast of rice production and consumption by 2030

Forecasting is a means for better understanding the effort needed to meet rice self-sufficiency goals. To align with the SDG period, which is also the target year of the second phase of CARD, the forecast horizon of 2030 was chosen. The ARIMA model was also used to forecast the annual rice consumption by 2030 using the historical trend from 1960 to 2018.

Forecasting rice production by 2030 is based on the potential area and attainable yield. In terms of the area, there is still a considerable amount of suitable land for rice cropping. The potential area is estimated to be more than 190 million ha (MHa) of inland valley in SSA (Deininger et al., 2011, Rodenburg et al., 2014), of which approximately 12% was used in 2018 (USDA, 2019)^4^. From this, we estimated the potential area for rice in the CARD countries to be more than 101 million ha. Yield gaps exists in many African countries, and yield represents, on average, 40% of its potential in 2018 (van Oort et al., 2015). The attainable yield would be on average 6 t/ha, with 8 t/ha, 6 t/ha and 4 t/ha in irrigated, lowland and upland ecology, respectively (Seck et al., 2010). We forecasted rice production based on the potential yields and area in the CARD countries.

To predict rice production, we employed three scenarios based on uses of area and potential yield levels. The first scenario of future rice production assumed that the average growth rate in yield and area in CARD countries over the period 2008–2018 would continue. This is equivalent to 2.6% and 4.5% growth in yield and area, respectively, and represents the baseline. The second scenario was an optimistic scenario and corresponded to the additional effort required by the second phase of the CARD to boost rice production to achieve self-sufficiency by 2030. The estimation shows that an annual increase in yield of 3% and area of 5.5% would be needed. This is equivalent to an increase of yield and area growth rates by approximately 20% compared to the baseline. The third scenario was a pessimistic scenario related to a decline in rice productivity due to yield limiting and reducing factors (higher incidence of pests, diseases, droughts, floods and climate change) and socioeconomic issues such as the current corona virus disease (COVID-19) pandemic. This scenario supposed a decrease of yield and area growth rate by half compared to the baseline, representing 1.3% and 2.25% growth in yield and area, respectively. Rice is grown in various environments (mainly upland, rainfed lowland and irrigated), but due to data availability, the analysis could not be performed for each environment. The analysis was performed for the combination of all production environments.

### 3.4 Data

The data used are from the United States Department of Agriculture (USDA) and FAO database (FAOSTAT), which are the two largest available databases on agricultural production in the world. The main database used was the USDA because it entirely covered the period of interest from 2008 to 2018^5^. In addition, the FAOSTAT was used for four countries (Ethiopia, Zambia, the Central African Republic and Rwanda). The production data for the Central African Republic and Ethiopia were not available from the USDA, while the data for Zambia and Rwanda were available only up to 2013 from the USDA.

## 4 Results

### 4.1 Trends in rice production from 1996 to 2018

**Fig. 2:**
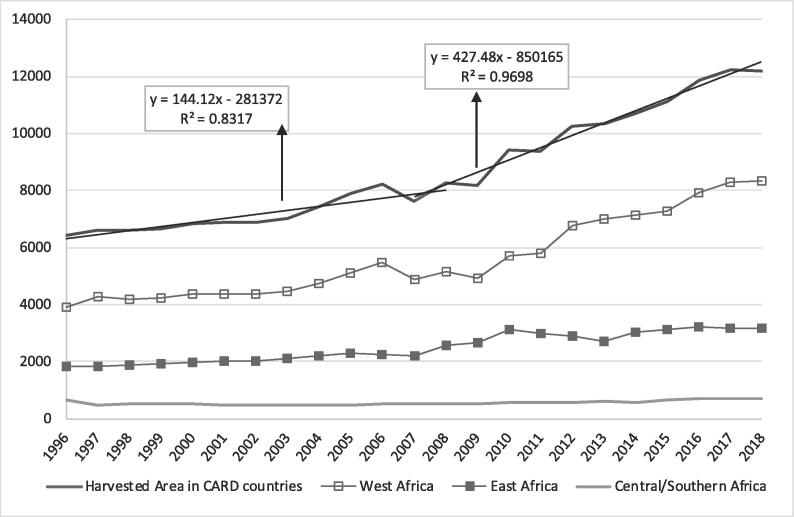
Trend in rice harvested area in the CARD countries (1000 ha).

This section compares the rice production statistics (harvested area, yield and production) trends between the decades before and after the 2007–2008 food crisis (1996–2007 and 2008–2018). A continuous increase in rice harvested area was observed in the CARD countries from 1996 to 2018 (Fig. 2). However, a higher slope in the rice harvested area was observed after 2008 and was mainly driven by West Africa^6^. During the decade before 2008, the harvested area increased by 19% (6.4–7.6 MHa from 1996 to 2007) in contrast to the 60% increase (7.6–12.2 MHa) between 2008 and 2018. Regional analysis revealed that West Africa had the highest increase of 70% (4.9 to 8.3 MHa), followed by East Africa at 44% (2.2–3.2 MHa) and Central/Southern Africa at 35% (0.5–0.7 MHa).

**Fig. 3:**
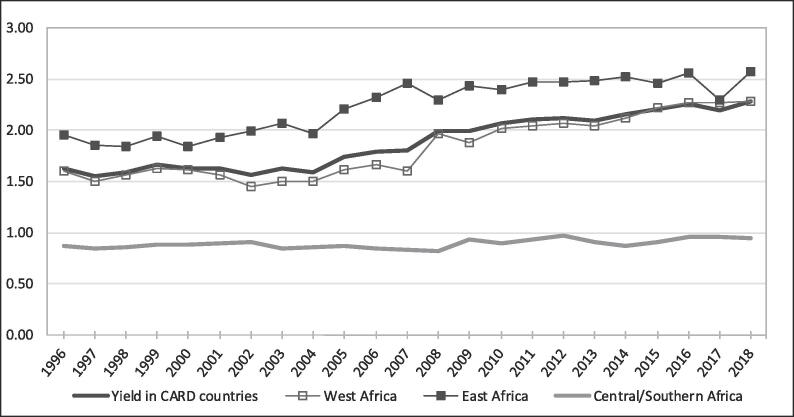
Trend in rice yield in the CARD countries (t/ha).

**Fig. 4:**
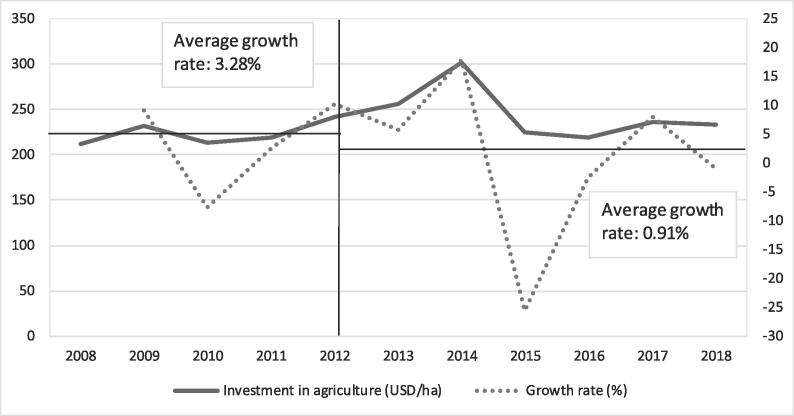
Trend of public agricultural expenditures and their growth rate from 2008 to 2018 (USD/ha) Source: https://www.resakss.org/ (accessed 17 July 2020).

The yield increased more rapidly during the 2008–2018 decade but was less than the area increase (Fig. 3). From 2008 to 2018, the yield increased by 27% (1.80 to 2.28 t/ha), in contrast with the only 10% increase before the food crisis (1.63 to 1.80 t/ha from 1996 to 2007). In other words, the yield increased by 1.3-fold in the CARD period, while the yield increased by only 1.1-fold in the previous decade. However, the yield growth rate after 2008 was not sustainable. The yield trend can be divided into two periods after 2008. From 2008 to 2012, the yield increased by 1.47% annually. From 2012 to 2018, the increase was only 1.19% annually. Country data analysis showed that 14 (61%) countries (including Benin, Cameroon, Cote d’Ivoire, Democratic Republic of Congo, Ethiopia, Kenya, Central African Republic, Nigeria, Rwanda, Senegal, Sierra Leone, Tanzania, Uganda and Zambia) followed this global trend of yield growth rate declining after 2012 (Table 1). Nine countries (Burkina Faso, The Gambia, Ghana, Guinea, Liberia, Madagascar, Mali, Mozambique, and Togo), however, showed continuous increased yield growth between the two periods. Using annual public expenditure in agriculture as a proxy of annual expenditure in the rice sector, we found that the growth rate of investment in agriculture per hectare decreased from 3.28% between 2008 and 2012 to 0.91% after 2012 (Fig. 4). This trend shows that public investment in agriculture was not sustainable after the food crisis, which had negative impact on the yield of crops such as rice.

**Table 1:** Yield growth rate during the periods 2008–2012 and 2012–2018 (%).

	Average yield growth rate 2008–2012 ^a^	Average yield growth rate 2012–2018 ^a^
Decreasing yield growth rate		
Benin	0.15	−1.64
Cameroon	9.63	−0.86
Central African Republic	18.92	0.87
Cote d’Ivoire	6.16	1.48
Democratic Republic of Congo	0.33	−0.77
Ethiopia	8.76	0.01
Kenya	33.69	0.14
Nigeria	2.08	0.55
Rwanda	6.52	−6.49
Senegal	5.18	−1.36
Sierra Leone	2.69	−1.27
Tanzania	9.19	3.63
Uganda	11.28	−0.45
Zambia	5.68	−4.53

Increasing yield growth rate		
Burkina Faso	−1.25	2.67
The Gambia	−6.94	−6.60
Ghana	2.85	3.25
Guinea	−0.13	1.23
Liberia	−4.05	2.30
Madagascar	−0.91	−0.34
Mali	−1.84	6.37
Mozambique	−18.52	−0.13
Togo	−8.30	−5.71

^a^Average annual yield growth rate was calculated using geometric mean.

Among the different regions, the East African countries had the highest yield level but the smallest yield increase of 5% (2.46 to 2.57 t/ha from 2008 to 2018), while Central/Southern Africa had the lowest yield level and the highest yield growth rate of 15% (0.82 to 0.94 t/ha from 2008 to 2018).

**Fig. 5:**
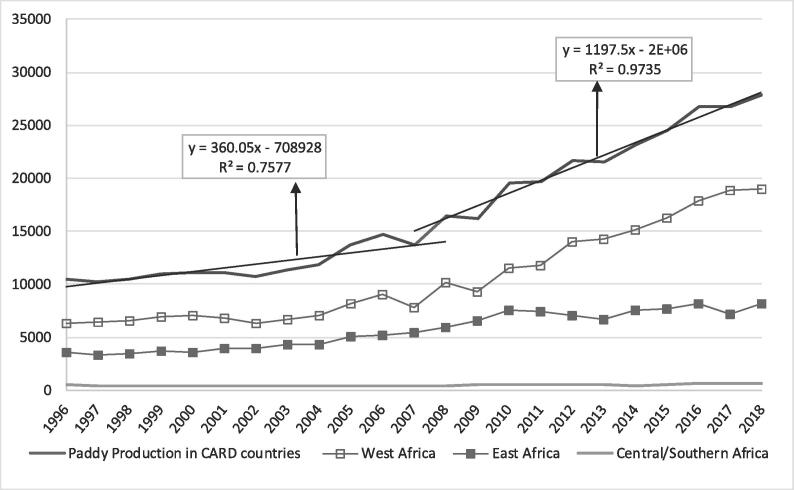
Trend in paddy production in the CARD countries (1000 tons).

Similar to the harvested area and yield, the rice production showed a continuous increase since 1996 (Fig. 5). However, the increase was the highest over the last decade in the CARD participating countries. Rice production increased by 103% (13.7 to 27.9 MT from 2008 to 2018) in contrast with an increase of 31% from 1996 to 2007. This difference is also shown by the higher slope of the linear trends of production during the period 2008–2018 (Fig. 5). Rice production increased by 2.03-fold during the CARD period. This trend aligned with the overall CARD objective of doubling rice production in SSA between 2008 and 2018. However, the achievement varied among regions and countries. West Africa showed an increase of 143% (7.8–19.0 MT); Central/Southern Africa, 54% (0.4–0.7 MT); and East Africa, 50% (5.5–8.2 MT). Only 7 countries (Cameroon, Ghana, Kenya, Senegal, Cote d’Ivoire, Benin and Tanzania) achieved the CARD objective of doubling rice production by 2018. However, the comparison of before and after the food crisis data may not reveal the real impact of the CARD. Without the CARD, trends in rice statistics would have changed over the decades, and the before-after comparison biases the estimation of the effect of the CARD. The next section will attempt to more robustly estimate the contribution of the CARD to rice production growth in the 23 countries.

### 4.2 Impact of the CARD policy

#### 4.2.1 Impact on rice harvested area and yield

**Fig. 6:**
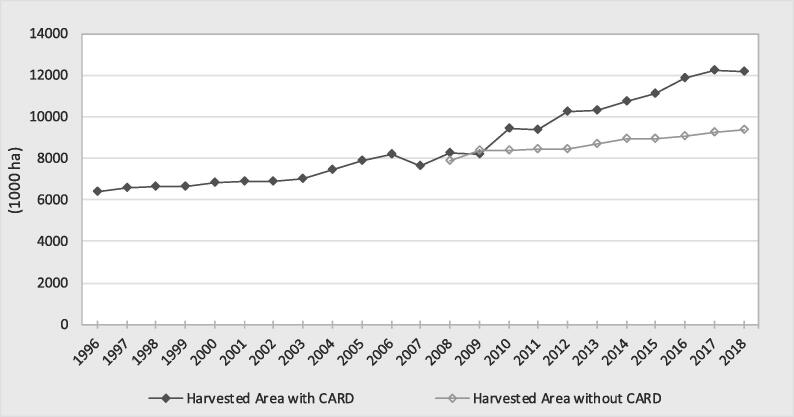
Comparison of the harvested area with the CARD and the counterfactual scenario.

Using the ARIMA model, the counterfactual scenarios (the trends of the harvested area and yield if there had been no CARD policy framework) were computed^7^ to estimate the real impact of the CARD. The results showed that the estimated impact of the CARD on rice harvested area was, on average, 1.7 MHa per year (Fig. 6 and Table A2 in the Appendix). The impact has continuously increased over time, especially since 2010, and the cumulative impact on the area was estimated at 18.1 MHa over the period 2008–2018. In 2018, the impact on area was 2.8 MHa, equivalent to 23% of the observed area.

**Fig. 7:**
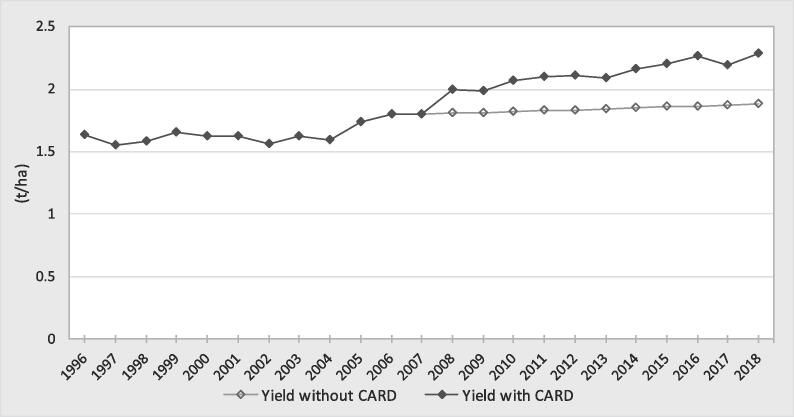
Comparison of the yield with the CARD and the counterfactual scenario.

Although the trend was different, a similar positive impact was estimated for the yield (Fig. 7). The impact on yield was 0.29 t/ha per year (Table A2 in the Appendix). However, the impact on yield varied from year to year. The highest impact was 0.41 t/ha in 2018, representing 19% of observed yield.

#### 4.2.2 Impact of the CARD on paddy production and self-sufficiency

**Fig. 8:**
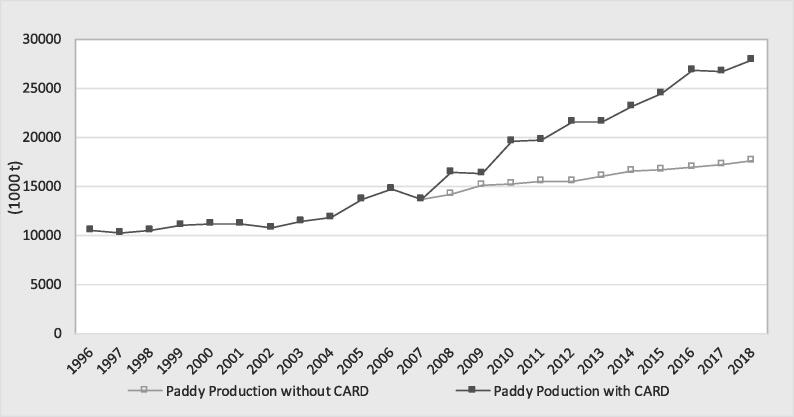
Comparison of paddy production with the CARD and the counterfactual scenario.

**Fig. 9:**
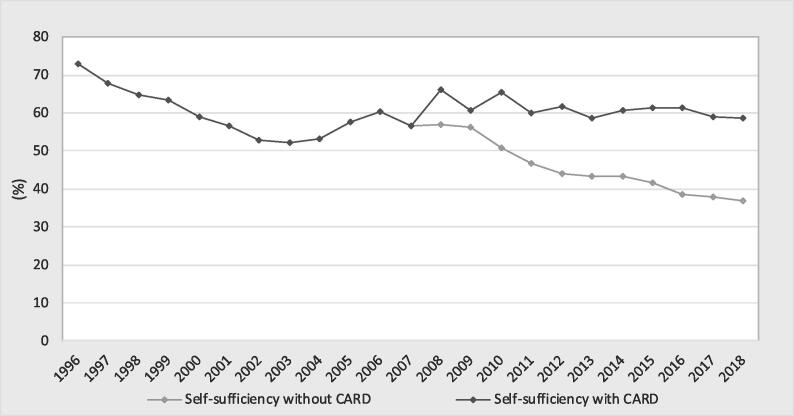
Comparison of self-sufficiency with the CARD and the counterfactual scenario.

Fig. 8, Fig. 9 show the trends in the actual production and rice self-sufficiency values and the counterfactual scenarios, respectively. The counterfactual values of production were below the observed values, meaning that the impact of the CARD on production was positive, and consequently, the effect on rice self-sufficiency was also positive. The annual impact of the CARD on production was estimated at 6.2 MT of paddy rice, on average (Table A2 in the Appendix). The cumulative impact of the CARD was estimated to be 67.7 MT of paddy production over the period 2008–2018. However, compared with the counterfactual situation, the contribution of the CARD in 2018 was 10.2 MT of paddy rice (the difference between 27.9 MT and 17.6 MT)^8^ compared to a target of 14 MT, representing a real achievement of 74%. The CARD policy in response to the 2008 food crisis had a positive impact on rice self-sufficiency. It helped to slow the increase in rice dependency in the 23 countries. Indeed, the CARD had an average contribution of a 16% increase in rice self-sufficiency per annum (Table A2 in the Appendix). For example, rice self-sufficiency would have been 37% compared to the observed value of 59% in 2018.

**Fig. 10:**
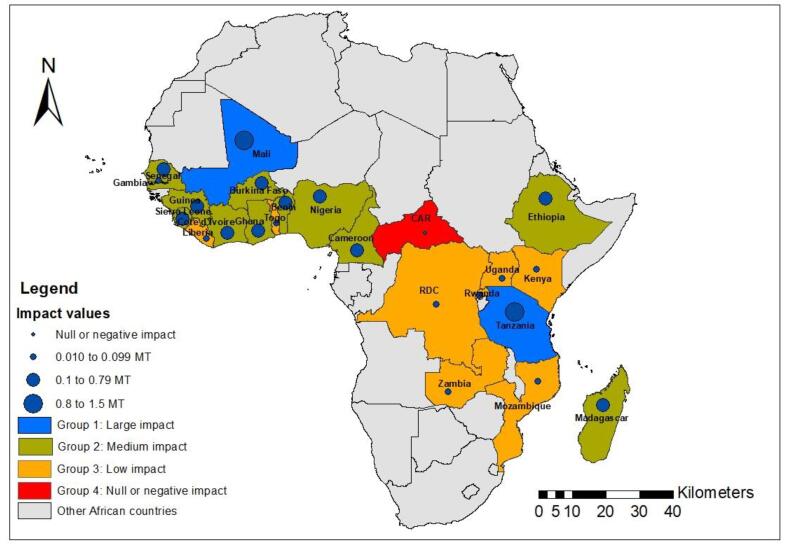
Grouping of countries based on CARD impact on production.

The impact of the CARD in each country was also estimated. The average impact over the period 2008–2018 varied among countries. Using the estimated impact on production, the countries can be categorized into four groups: large positive impact (0.8 to 1.5 MT), medium positive impact (0.1 to 0.79 MT), low positive impact (0.01 to 0.09 MT) and null or negative impact countries. The first group included two countries (Tanzania and Mali). The second group comprised 11 countries (Cote d’Ivoire, Nigeria, Senegal, Ghana, Sierra Leone, Guinea, Benin, Burkina Faso, Madagascar, Ethiopia and Cameroon) (Fig. 10 and Table A3 in the Appendix). Nine countries (Liberia, Mozambique, The Gambia, Togo, Congo, Kenya, Rwanda, Uganda and Zambia) were classified into the third group, and one country (the Central African Republic) was in the fourth group. Impact at the country level revealed that the higher the production in a country was, the higher the impact on total production. However, when estimating the relative production increase due to CARD (Table A3 in the Appendix), the performances of Tanzania and Mali in the first group (50% and 42%, respectively) were lower than those in Mozambique and Zambia in the third group (98% and 66%, respectively).

#### 4.2.3 Determinants of CARD impact on rice production

Determinants of CARD impact in countries were investigated using simple ordinary least square regression. The model is globally significant at 5%, and the R-squared is high (64%). Robust standard errors are reported to avoid any heteroskedasticity problem. The variance inflation factors (VIFs) showed that multicollinearity is not a problem in the model.

The results showed that investments in demand‐pull factors have a stronger effect on the contribution of CARD to production than do investments in supply‐push factors (Table 2). Marginal effects showed that countries with high investments in value-chain upgrading have obtained 0.45 MT per year more than countries with no evidence of investments in value-chain upgrading. This effect is 0.15 MT per year for country with moderate investments in value-chain upgrading. The coefficient of the coastal countries with preference for local rice is positive and significant. Thus, the impact of the CARD was higher in coastal countries with preference for local rice than in landlocked countries. The coefficient of number of varieties released or adopted was also significant at the 1% level. However, its marginal effect (0.018 MT of paddy rice per year) was lower than the marginal effect of three variables related to demand-pull factors. Moreover, the effects of two other supply-push factors (quantity of fertilizer per hectare and the share of irrigated area) were not significant. This result showed the relative importance of demand-pull investments compared to supply-push investments for achieving rice self-sufficiency.

**Table 2:** Determinants of cumulative impact of CARD on rice production over 2008–2018.

Variables	Coefficients	Robust standard errors
Number of varieties release or adopted (number)	182.48***	58.28
Fertilizer used per hectare (kg/ha)	40.82	39.91
Share of irrigated area (%)	−29.06	18.46
Number of extension agents (number)	0.00	0.05
Moderate value-chain upgrading investment (dummy)^ϒ^	1504.71**	697.73
High value-chain upgrading investment (dummy)^ϒ^	4478.37*	2459.40
Coastal countries with preference for local rice (dummy)^Ϯ^	2959.13*	1530.67
Coastal countries with preference for imported rice (dummy)^Ϯ^	836.48	1233.38
Constant	−2893.75*	1492.95

*Notes*: Sample size = 22; R-squared = 64%. The p-value of Fisher test (F = 2.61) was 0.06. The variance inflation factors (VIFs) are in the range of 1.15–1.89, with a mean VIF of 1.44. Significance levels: *p < 0.1; **p < 0.05; ***p < 0.01. Data on fertilizer use per hectare are from https://www.resakss.org/ (accessed 17 July 2020). For data on number of varieties released and share of irrigated area, we exploited information in Diagne et al., 2013, Saito et al., 2015 and https://strasa.irri.org/varietal-releases (accessed 17 July 2020). ^ϒ^ Investment in value-chain upgrading is based on the groups defined by Soullier et al. (2020), and the third group of “no evidence of upgrading investment” is the reference group. ^Ϯ^ The reference group is “Landlocked country” following Demont (2013). The dependent variable is the cumulative impact of the CARD per country and is expressed in 1000 tons.

### 4.3 Rice production and self-sufficiency by 2030

**Fig. 11:**
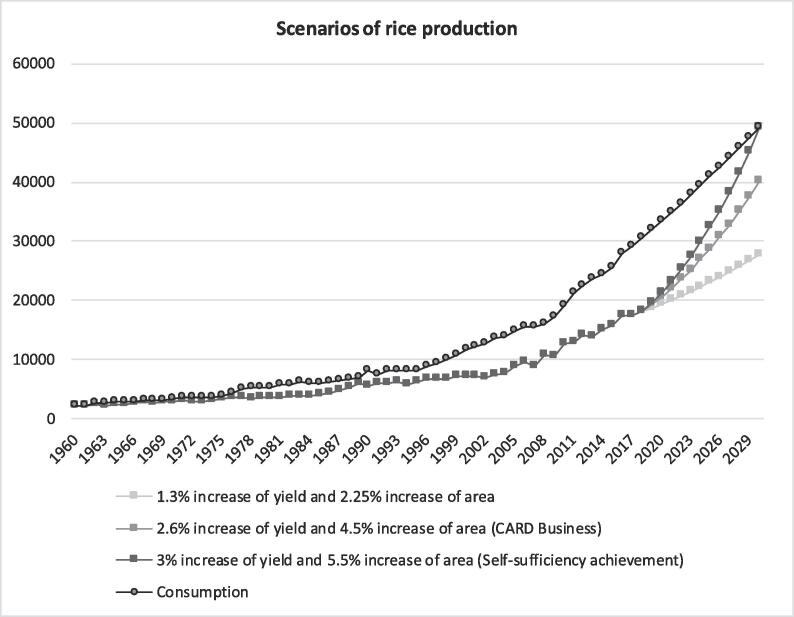
Forecast rice production and consumption by 2030 (1000 tons of milled rice).

Rice consumption was forecasted by 2030 using the ARIMA model (Fig. 11)^9^. The results showed that the consumption was expected to reach approximately 49.2 MT of milled rice by 2030 in the 23 countries^10^ compared to a total consumption of 30.6 MT of milled rice in 2018.

We developed scenarios for production by 2030. With the first scenario being business as usual (the baseline), with an annual increase in yield of 2.6% and area of 4.5%, the total production would be approximately 40.1 MT by 2030. This would lead to a consumption-production gap of approximately 9 MT of milled rice to be imported from Asia or the Americas. This would likely cost approximately US$ 5.8 billion per year. To reduce this importation bill and achieve rice self-sufficiency, an optimistic scenario was assessed (Fig. 11). The optimistic scenario with a high annual increase in yield of 3% and area of 5.5% (increase of approximately 20% of yield and area growth rates compared to the baseline) would result in a production equivalent to 49.2 million tons of milled rice and would help to achieve self-sufficiency by 2030. This is theoretically achievable because this scenario is equivalent to the use of 54% of the potential yield and 23% of the potential area by 2030 compared to the 40% and 12% of the potential yield and area in use, respectively, in 2018. The low-increase scenario (pessimistic scenario) supposed that yield and area would increase less than what was observed in the last decade (1.3% and 2.25% growth rates in yield and area, respectively). In this scenario, production would be approximately 27.6 MT of milled rice, leading to 44% import dependency.

## 5 Discussion

The 2007–2008 volatility of rice prices and its aftermath on food security in SSA have led to urgent policy responses, including the CARD. The objective of the CARD was to double domestic production, i.e., from 14 MT to 28 MT of paddy rice by 2018. The results showed that rice production increased faster during the decade of the implementation of the CARD than during the decade before. Rice production increased by 103% from 2008 to 2018 in contrast with an increase of only 31% from 1996 to 2007. These results confirmed the findings of other studies (Seck et al., 2010, Saito et al., 2015). Seck et al., 2010, Saito et al., 2015 also found that rice production increased faster after the food crisis in 2008 than in the previous period. However, the yield growth was not sustainable throughout the CARD period. From 2008 to 2012, the yield increased by 1.47% per year, while it was only 1.19% from 2012 to 2018. Fourteen countries out of 23 showed this trend. This result complements the findings of Saito et al. (2015), who observed that between 2007 and 2012, the rice yield per hectare almost doubled, and the rice production increase was facilitated by yield growth rather than by area expansion. While the yield increased between 2008 and 2012, the growth rate declined between 2012 and 2018. The decline in the yield growth rate was the result of the inability of the policy measures to sustain the yield growth. During the CARD, the main actions were supply-push measures such as free seed distribution, subsidies of fertilizer and irrigation rehabilitation schemes through development projects implemented in the countries. However, these actions responded to emergency of the food crisis and stopped at the end of the projects. This limitation was confirmed by the declining of investments during the CARD policy period. The growth rate of investment in agriculture per hectare decreased from 3.28% between 2008 and 2012 to only 0.91% between 2012 and 2018 despite the commitment of African leaders to allocate at least 10% of the total government expenditure to the agriculture sector. Sustainable yield growth requires long-term development actions (Binswanger and Deininger, 1997).

The impact assessment of the CARD showed that over the period of implementation, 74% of the objective of doubling rice production in 2018 was achieved. The results showed that the contribution of CARD is significantly determined by investments in demand-pull factors through investments in value-chain upgrading. The higher the investments in value-chain upgrading, the higher the impact of CARD policy. This finding suggests that investments in value-chain upgrading through modern mill development and vertical coordination were able to increase the rice paddy production. Modern mills require high quantities of paddy rice to reach profitability and to recover the investments. Therefore, modern mill owners also invested directly in rice production through vertical integration or indirectly through contract farming, which was also found to have a positive impact on production (Arouna et al., 2019). This result confirms the findings of Demont (2013), who argued that more resources need to be provided for value addition and demand-pull investment in the rice sector in SSA. However, value-chain upgrading is still marginal in SSA. Soullier et al. (2020) found that only Senegal and Nigeria can be considered dynamic in value-chain upgrading in West Africa during the CARD period. As was the case in Senegal and Nigeria, value-chain upgrading in SSA should be led by the private sector for sustainability and efficiency in management. Due to the low share of demand-pull investments in the first generation of the NRDSs in many countries (Demont, 2013), more resources will need to be allocated to private-led value-chain upgrading during the second phase of the CARD to achieve self-sufficiency by 2030. The results show that impact of the CARD was higher in coastal countries with dominant preference for local rice, while the impact was not significant in coastal countries with dominant preference for imported rice. Therefore, investment in the rice sector in coastal countries with dominant preference for local rice and landlocked countries will have a higher impact than that in coastal countries with dominant preference for imported rice. Coastal countries with dominant preference for imported rice will need to invest more and may also need more time to achieve self-sufficiency in rice production. This situation can be explained by the fact that local rice is not a perfect substitute for imported rice in countries with dominant preference for imported rice (Demont, 2013).

The results showed that among supply-push factors, only the number of high-yielding varieties released and adopted has a positive effect on the impact of the CARD. Improved high-yielding varieties are important not only for productivity growth but also for the adaptation of cropping systems to climate change and other stresses (iron toxicity, salinity, etc.) (van Oort, 2018). Arouna et al., 2017 showed that improved rice varieties positively affect productivity and production. Surprisingly, the effect of inorganic fertilizer on production growth during the CARD period was not significant. This finding may be explained by two main factors. First, SSA is characterized by low use of fertilizer (Sheahan and Barrett, 2017, MacCarthy et al., 2018) in conjunction with bush burning, residual removal from the field, reduction in area of fallow fields, high levels of deforestation, land degradation and nutrient depletion, indicating unsustainable land use. Although there is heterogeneity among countries and within countries regarding the use of fertilizer, the current level of inorganic fertilizer use cannot allow farmers to realize the potential yield gain. The efficiency of inorganic fertilizers requires the use of organic fertilizers and improved germplasm along with good agricultural practices (Vanlauwe et al., 2015) as well as water management. Second, blanket fertilizer recommendations are the general approach in many countries in SSA. Blanket fertilizer recommendations do not consider the variation in local settings but, rather, are uniform in space and time (MacCarthy et al., 2018). Failure to formulate fertilizer recommendations that are soil and crop specific and that consider the effect of climate variability results in inefficiency in fertilizer use. Therefore, the use of inorganic fertilizer based on blanket recommendations throughout the CARD period hinders the achievement of the expected yield gain. However, new applications such as RiceAdvice or Crop Manager that can deliver target recommendations are increasingly available and are expected to increase the efficiency of inorganic fertilizer (Arouna et al., 2020).

Share of irrigated area has no effect on the impact of the CARD. Many investments in irrigation schemes have failed to deliver the anticipated benefits (Byiringo et al., 2020). Many schemes fail due to a lack of collective action over basic maintenance issues and absence of a coordination mechanism to allocate water across users in the system. In addition, inefficient irrigation systems are major problems in rice irrigation ecology (Fahad et al., 2019). Therefore, sustainable rice production in existing irrigation schemes requires collective action and coordination for maintenance.

To match consumption by 2030, production needs to increase by 2.7 times the 2018 level, indicating that an important investment will be needed to achieve rice self-sufficiency by 2030. Policy measures leading to annual increases in yield and area by 3% and 5.5%, respectively, would allow self-sufficiency to be achieved by 2030^11^. Most African governments are implementing the National Agriculture Investment Plan (NAIP) and the second phase of the CARD. However, to achieve rice self-sufficiency, it is important to identify the proper areas for investment. Priority area of interventions should be investments in private-led value-chain upgrading through modern mill development and contract farming as well as the release of improved varieties and seed system development. AfricaRice (2018) has developed models for back-of-the-envelope calculations of investment to achieve rice self-sufficiency, which can be used by policy makers. We recognize, however, that ecology-specific recommendations would be more useful if data were available to analyze the contribution of the CARD for specific growing environments.

## 6 Conclusion

This study assessed the contribution of the CARD through the analysis of the situation of rice production before and after the CARD implementation and the estimation of the impact of the CARD on the rice harvested area, yield, production and self-sufficiency using a combination of the ARIMA model and the counterfactual framework. The results indicated that rice production increased more during the CARD decade than during the previous decade. The CARD increased rice production in participating countries. The experience of the CARD revealed that policy measures designed and implemented effectively will generate progress in rice sector development for self-sufficiency and food security in SSA. However, yield growth was not sustainable throughout the CARD period due to the relaxation in government investment after 2012.

Although local rice production increased rapidly after the 2008 food crisis, it has never caught up with demand. Considering the results of the CARD, the goal of self-sufficiency achievement requires policy measures to be implemented in a sustained and efficient manner and over the long term. Scenario analysis showed that annual increases of 3% and 5.5% in yield and area, respectively, would lead to the achievement of self-sufficiency by 2030. Based on the lessons learned from the CARD, value-chain upgrading through private investments in the modern milling sector as well as operational vertical coordination should be the priorities for sustainable rice production growth to achieve rice self-sufficiency in SSA.

## CRediT authorship contribution statement

**Aminou Arouna:** Conceptualization, Methodology, Writing - original draft, Writing - review & editing. **Irene Akoko Fatognon:** Investigation, Data curation, Formal analysis. **Kazuki Saito:** Visualization, Writing - review & editing. **Koichi Futakuchi:** Writing - review & editing.

## Declaration of Competing Interest

The authors declare that they have no known competing financial interests or personal relationships that could have appeared to influence the work reported in this paper.

## Figures and Tables

**Fig. A1:**
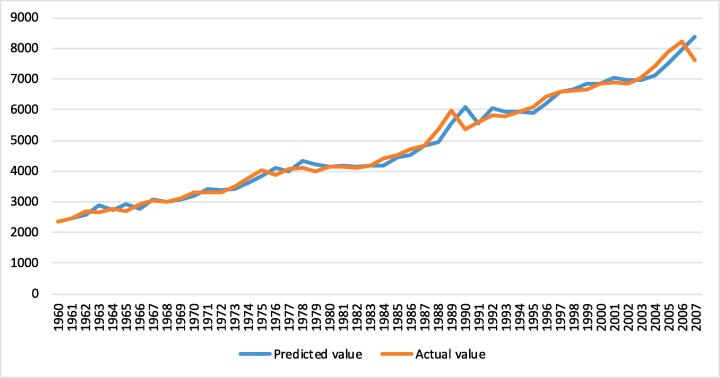
Validity of the ARIMA model of area prediction.

**Fig. A2:**
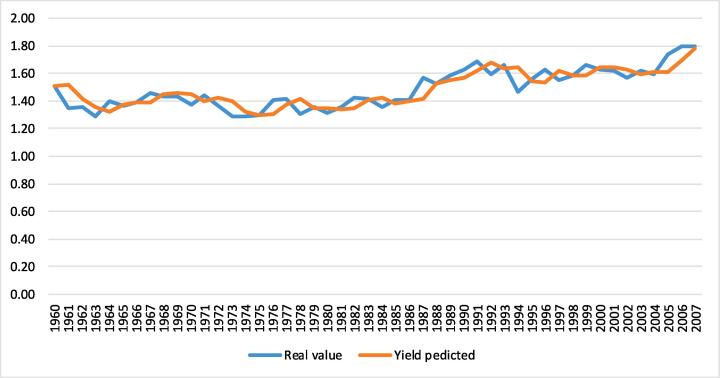
Validity of the ARIMA model of yield prediction.

**Fig. A3:**
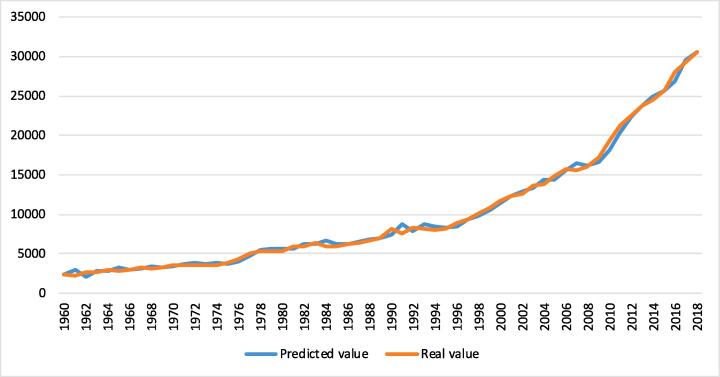
Validity of the ARIMA model of consumption prediction.

**Table A1:** Classification of countries by status of value-chain upgrading and preference for local or imported rice.

	Status of value-chain upgrading (1 = High value-chain upgrading investment; 2 = Moderate value-chain upgrading investment, 3 = no evidence of upgrading investment)*	Status of geographic situation and preference for local or imported rice (1 = Coastal countries with preference for imported rice; 2 = Coastal countries with preference for local rice; 3 = Landlocked country)**
Benin	2	1
Burkina Faso	2	3
Central African Republic	3	3
Cameroon	2	1
Cote d'Ivoire	2	1
Congo	3	1
Ethiopia	1	3
The Gambia	3	1
Ghana	2	1
Guinea	3	2
Kenya	3	2
Madagascar	2	2
Mali	2	3
Mozambique	1	2
Nigeria	1	1
Rwanda	3	3
Senegal	1	1
Sierra Leone	2	2
Tanzania	1	2
Togo	2	1
Uganda	2	3
Zambia	2	3

*Source*: * Based on Soullier et al. (2020) for West African countries and expert opinion for other countries. ** Based on Demont (2013) for West African countries and expert opinion for three countries (Congo, The Gambia and Central African Republic).

**Table A2:** Estimates of the annual impact of the CARD policy.

Year	2008	2009	2010	2011	2012	2013	2014	2015	2016	2017	2018	Average annual impact
Harvested area (1000 ha)	380	−170	1047	936	1765	1632	1796	2153	2803	2986	2810	1649
Yield (t/ha)	0.19	0.17	0.25	0.27	0.28	0.25	0.31	0.35	0.40	0.32	0.41	0.29
Production (1000 tons)	2228	1118	4262	4279	6081	5545	6648	7846	9921	9502	10,232	6151
Self-sufficiency (%)	9	4	14	13	18	15	18	20	23	21	22	16

**Table A3:** Estimation of CARD impact by country.

Countries	2008	2009	2010	2011	2012	2013	2014	2015	2016	2017	2018	Annual average impact
Large positive impact group												
Tanzania	320 (23)	307 (23)	892 (45)	1142 (51)	712 (40)	1126 (51)	1534 (59)	1875 (63)	2363 (69)	2036 (66)	2052 (66)	1305 (50)
Mali	427 (26)	731 (68)	1042 (80)	396 (23)	814 (42)	759 (34)	651 (30)	754 (32)	1133 (41)	1209 (41)	1112 (38)	821 (42)

Medium impact group												
Cote d'Ivoire	−117 ( −1 7)	−205 ( −3 0)	245 (20)	−328 ( −3 8)	330 (21)	531 (27)	608 (30)	1072 (50)	979 (48)	1027 (49)	1103 (49)	477 (19)
Nigeria	314 (8)	−583 ( −1 6)	205 (5)	199 (4)	69 (1)	−137 (−3)	−118 (−2)	1545 (25)	1212 (17)	1156 (16)	1075 (14)	449 (6)
Sierra Leone	69 (10)	329 (37)	457 (44)	556 (49)	564 (49)	673 (54)	142 (19)	281 (32)	567 (49)	801 (57)	594 (50)	458 (41)
Senegal	194 (48)	178 (46)	392 (65)	195 (48)	260 (55)	225 (52)	349 (62)	696 (77)	737 (78)	799 (79)	742 (78)	433 (63)
Ghana	56 (19)	136 (35)	256 (52)	210 (45)	218 (45)	307 (54)	332 (55)	363 (57)	404 (59)	442 (60)	553 (74)	298 (50)
Guinea	102 (7)	−2 (0)	16 (1)	159 (10)	382 (20)	488 (24)	378 (19)	430 (21)	528 (24)	524 (24)	573 (25)	325 (16)
Burkina Faso	133 (68)	147 (69)	196 (72)	158 (65)	245 (77)	242 (79)	286 (82)	256 (79)	311 (81)	252 (77)	306 (82)	230 (76)
Cameroon	2 (4)	69 (52)	88 (58)	107 (61)	112 (61)	121 (62)	129 (85)	204 (74)	282 (79)	273 (78)	352 (77)	158 (63)
Madagascar	327 (8)	937 (21)	1120 (24)	677 (16)	922 (20)	−24 (−1)	340 (9)	87 (2)	182 (5)	−430 ( −1 4)	167 (4)	392 (9)
Ethiopia	61 (85)	93 (90)	80 (89)	79 (89)	111 (92)	83 (90)	123 (93)	132 (94)	129 (95)	134 (95)	138 (96)	106 (92)
Benin	21 (19)	32 (28)	36 (29)	132 (60)	127 (58)	108 (52)	138 (59)	100 (49)	179 (64)	170 (61)	169 (60)	110 (49)

Low positive impact group												
Liberia	48 (17)	54 (18)	58 (20)	76 (26)	71 (24)	50 (19)	45 (19)	54 (19)	75 (28)	62 (22)	59 (20)	59 (21)
Mozambique	14 (7)	−29 ( −1 6)	61 (24)	74 (27)	−47 (−32)	157 (45)	157 (41)	171 (48)	145 (44)	183 (46)	187 (48)	98 (26)
Gambia	12 (32)	51 (65)	74 (73)	23 (45)	27 (50)	44 (62)	23 (48)	27 (52)	34 (69)	8 (26)	25 (56)	32 (52)
Togo	6 (7)	41 (34)	31 (28)	31 (28)	81 (50)	73 (46)	65 (45)	61 (43)	56 (41)	62 (44)	60 (43)	52 (37)
Kenya	−38 ( −1 6 5)	−14 (−33)	23 (27)	52 (47)	62 (45)	31 (25)	45 (40)	57 (49)	58 (49)	60 (50)	60 (50)	36 (17)
Congo	2 (1)	3 (1)	11 (3)	11 (3)	42 (13)	47 (15)	52 (17)	−9 (−3)	−9 (−3)	−9 (−3)	−9 (−3)	12 (4)
Zambia	8 (35)	29 (69)	37 (72)	37 (74)	33 (73)	32 (71)	37 (75)	14 (55)	16 (60)	26 (69)	32 (74)	27 (66)
Uganda	10 (6)	41 (20)	54 (25)	67 (29)	45 (21)	45 (21)	65 (27)	62 (26)	70 (28)	64 (26)	61 (25)	53 (23)
Rwanda	17 (20)	13 (16)	−3 (−5)	7 (9)	8 (10)	15 (16)	−8 ( −1 2)	14 (14)	24 (22)	20 (18)	28 (23)	11 (12)

Negative impact group												
Central African Republic	−4 ( −1 0)	−7 ( −1 7)	−14 ( −3 6)	−20 ( −4 9)	−17 ( −4 0)	−37 ( −1 2 5)	−51 ( −4 0 0)	−58 ( −5 7 2)	−58 ( −5 0 1)	−62 ( −4 7 8)	−71 ( −6 4 3)	−36 ( −2 6 1)

*Note*: () Percent of increase of impact; production is expressed in 1000 tons.

## Footnotes

1 https://riceforafrica.net/

2 CARD activities were coordinated and backstopped by a secretariat. However, CARD evaluation in this paper is not related to the evaluation of the CARD secretariat activities, and the investment is not related to the CARD secretariat.

3 One might consider the use of data from nine new countries in CARD phase 2 or from other similar countries as counterfactual conditions. However, either of these approaches would yield biased counterfactual situations for two main reasons. First, the fact that nine countries did not join the CARD phase 1 implies that they are different from CARD phase 1 participating countries. Their values cannot be used as counterfactual data. Second, countries are different both in rice production and in production trends. Therefore, the best approach is to use historical data of participating countries to estimate the counterfactual situation.

4 The potential area for rice cropping is far greater than the 190 MHa of inland valleys because rice is also grown upland.

5 At the time of the data analysis, the 2018 data were not available on FAOSTAT.

6 The regional distribution of CARD countries in SSA is given as follows: West Africa: Benin, Burkina Faso, Cote d’Ivoire, The Gambia, Ghana, Guinea, Liberia, Mali, Nigeria, Senegal, Sierra Leone and Togo. East Africa: Ethiopia, Kenya, Uganda, Rwanda and Tanzania. Central Africa: Democratic Republic of Congo, Central African Republic and Cameroon. Southern Africa: Mozambique and Zambia.

7 ARIMA (4, 2, 0) and ARIMA (1,1,0) had the best fits for the harvested area and yield, respectively. Fig. A.1. and Fig. A.2. in the Appendix show that these two models fit the data well.

8 The rice production in the 23 participating countries was 27.9 MT in 2018, but the production would have been 17.6 MT without CARD.

9 ARIMA (1,2,1) was the best fit for rice consumption (Fig. A.3. in the Appendix).

10 Scenarios concern the 23 countries in the first phase of CARD. The new countries (Angola, Burundi, Chad, Congo, Gabon, Guinea Bissau, Malawi, Niger and Sudan) of the second phase of CARD are not included.

11 However, it is worth noting that increased production may affect consumption through the change in price. The effect of price was kept constant in our analysis, which is a limitation.
